# Breast MRI in Patients with Unilateral Bloody and Serous-Bloody Nipple Discharge: A Comparison with Galactography

**DOI:** 10.1155/2015/806368

**Published:** 2015-01-22

**Authors:** Lucia Manganaro, Ilaria D'Ambrosio, Silvia Gigli, Francesca Di Pastena, Guglielmo Giraldi, Stefano Tardioli, Marialuisa Framarino, Lucio Maria Porfiri, Laura Ballesio

**Affiliations:** ^1^Department of Radiological Sciences, Sapienza University of Rome, Umberto I Hospital, Viale Regina Elena 324, 00161 Rome, Italy; ^2^Department of Public Health and Infectious Diseases, Sapienza University of Rome, Piazzale Aldo Moro 5, 00185 Rome, Italy; ^3^Department of Gynecology and Obstetrics, Sapienza University of Rome, Umberto I Hospital, Viale Regina Elena 324, 00161 Rome, Italy

## Abstract

*Purpose*. Assessing the role of breast MRI compared to galactography in patients with unilateral bloody or serous-bloody nipple discharge.* Materials and Methods*. Retrospective study including 53 unilateral discharge patients who performed galactography and MRI. We evaluated the capability of both techniques in identifying pathology and distinguishing between nonmalignant and malignant lesions. Lesions BIRADS 1/2 underwent follow-up, while the histological examination after surgery has been the gold standard to assess pathology in lesions BIRADS 3/4/5. The ROC analysis was used to test diagnostic MRI and galactography ability.* Results*. After surgery and follow-up, 8 patients had no disease (15%), 23 papilloma (43%), 11 papillomatosis (21%), 5 ductal cancer in situ (10%), and 6 papillary carcinoma (11%) diagnoses. Both techniques presented 100% specificity; MRI sensitivity was 98% versus 49% of galactography. Considering MRI, we found a statistical association between mass enhancement and papilloma (*P* < 0.001; AUC 0.957; CI 0.888–1.025), ductal enhancement and papillomatosis (*P* < 0.001; AUC 0.790; CI 0.623–0.958), segmental enhancement and ductal cancer in situ (*P* = 0.007; AUC 0.750; CI 0.429–1.071), and linear enhancement and papillary cancer (*P* = 0.011).* Conclusions*. MRI is a valid tool to detect ductal pathologies in patients with suspicious bloody or serous-bloody discharge showing higher sensitivity and specificity compared to galactography.

## 1. Introduction

Nipple discharge represents about 7–10% of breast symptoms and it is the third most common complaint after pain and breast masses [1–4]. It is usually caused by benign conditions, but occasionally it indicates a serious medical problem, especially when it is spontaneous and bloody/serous-bloody and is not associated with physiological conditions, such as pregnancy or breastfeeding [5].

The most common causes are some benign breast lesions [6], such as solitary intraductal papilloma and papillomatosis followed, in a small percentage of cases (between 5% and 21%), by malignant lesions, such as papillary carcinoma, ductal cancer in situ (DCIS), and invasive ductal carcinoma [7, 8].

The diagnostic patients management requires a complete clinical history, physical examination, cytological exam, and radiological exams [9]. Unfortunately, the cytological exam is not always diagnostic. Mammography and sonography are considered the first imaging methods performed as standard of care in the nipple discharge diagnostic evaluation. Nonetheless, both methods may present some limitations and difficulties in evaluating intraductal lesions [10, 11].

For a long time, galactography has been considered the gold standard to assess nipple discharge [12, 13]. It directly shows the secreting duct, which is cannulated and opacified through the contrast medium injection [14]. It is a safe and economical method, in most cases easily available. However, it does not always display specific findings, so it could be difficult to build a differential diagnosis between benign and malignant ductal breast diseases [15, 16].

Moreover, it could be difficult to cannulate the duct, especially in patients with intermittent discharge or nipple retraction. Another important detail is the ionizing radiation use, chiefly in up to 35 years old patients [17].

Magnetic resonance imaging (MRI) is a high sensitivity technique in detecting breast diseases such as invasive breast cancer (68–100%) and DCIS (77–96%) [18, 19].

Several studies have been conducted to evaluate the potential MRI diagnostic role in ductal pathologies diagnosis, but few researchers have made a comparison between MRI and galactography [9, 20–22].

The aim of the study is the comparison between galactography and MRI in patients with bloody/serous-bloody unilateral nipple discharge, in order to evaluate their sensitivity, specificity, and accuracy for the right differential diagnosis between benign and malignant diseases.

## 2. Materials and Methods

### 2.1. Members and Inclusion Criteria

In our study, we performed a retrospective analysis. We extrapolated patients presenting bloody or serous-bloody nipple discharge from our radiological database, which collected 1700 consecutive breast MRIs. Fifty-three female patients, aged between 28 and 65 years of age (average age 42 years old), respected our inclusion criteria. They presented unilateral bloody or serous-bloody nipple discharge and performed both galactography and MRI exams at our Radiological Sciences Department. The time elapsed between the two exams was two weeks maximum. All patients presenting lesions classified as BIRADS 1 or BIRADS 2 underwent follow-up, performed with mammography, ultrasound, or cytology. The average follow-up was 18 months. The histological examination has been the gold standard to assess the kind of pathology in lesions classified as BIRADS 3, BIRADS 4, and BIRADS 5.

### 2.2. Galactography

Galactography was performed cannulating the secretory duct with a blunt dedicated cannula, through which was injected a nonionic iodinated contrast agent (iopamidol 300) up to a maximum of 1–1.5 mL. After the cannula removal, radiographs on craniocaudal and mediolateral oblique projections have been acquired with a digital mammography system (Trade Art 2000, Planmed Nuance Aprilia, Rome).

**Figure 1:**
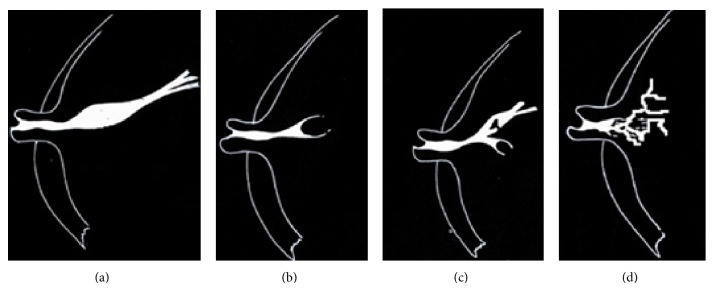
Image findings of (a) ductal ectasia, (b) filling stop, (c) filling defect, and (d) ductal distortion.

Findings were classified according to the Gregl scheme [23] into the following groups as showed in Figure 1:

1. technically inadequate investigation,
2. normal findings,
3. ductal ectasia (i.e., duct over 2 mm),
4. filling defects,
5. filling stops,
6. ductal distortions.

### 2.3. Magnetic Resonance Imaging

Magnetic resonance imaging (MRI) has been performed using a 1.5 Tesla magnet (Magnetom Vision, Avanto Siemens Medical System, Erlangen, Germany; gradients 25 mT/m^2^; slew rate 800 T/m/s; rise time 400 *μ*s) with dual coil dedicated to the breast study (4-Channel BI Breast Coil). In premenopausal patients we performed the examination between the 7th and the 14th menstrual cycle day.

The protocol includes sequences obtained before contrast medium administration: T2-STIR weighted sequences on the axial plane (TR 5320/TE 58 ms; FOV read 300; FOV phase 100; slice thickness 3.5 mm without gap, length 5 min); 3D Flash NFS T1-weighted sequences on the axial plane (TR 7.73/TE 4.76 ms; Flip angle 25, FOV read 320; FOV phase 100; thickness 1 mm; length 1 min and 37 s); and T1 Flash 3D FS on the sagittal plane (TR/TE 8/5 ms; FOV read 330; FOV phase 100; slice thickness 1 mm; length: 1 min and 50 s) on the secretory breast.

The image acquisition on axial and sagittal planes has allowed three-dimensional ductal tree visualization.

Then we used 3D gradient recalled echo (GRE) fat saturation T1-weighted sequences on axial and sagittal planes, acquiring a single scan before (length 2 min and 53 sec) and four consecutive scans (delay of 10 sec from basal scan) after contrast medium administration length of each scan (2 min and 53 sec without delay). Total length of the exam is about 20 minutes.

The contrast medium introduction was performed using an automatic injector administrating gadobutrol at a dose of 0.1 mmol per body weight kilogram with a rate of 2 mL/s together with 10 mL of saline bolus.

T2-STIR sequences were used to obtain information about breast morphology and structure (presence of fatty, fibrous, and glandular tissue) and to evaluate the ductal system. Ductal ectasia appeared in the shape of single or multiple tubular images thanks to high signal intensity on T2-weighted images.

Flash 3D fat saturation (FS) precontrast T1-weighted images were analyzed for intraluminal fluid content characterization, identifying the presence of high signal intensity, such as blood or proteinaceous material.

To evaluate any enhancing areas we executed T1-weighted sequences after contrast medium administration. In particular, we considered parameters like morphology and the type of enhancement. For each enhancing lesion, we classified the type of enhancement according to the BIRADS lexicon as mass-like or non-mass-like (linear, ductal, segmental, and regional).

Two radiologists of 15- and 5-year experience in breast imaging blinded to the histological examination independently reviewed MRI and galactographic exams.

### 2.4. Histopathology

Thirty-three patients underwent core-needle biopsy (CNB) procedure. CNB has been performed under ultrasound (US) guidance using a 14 Gauge automated biopsy gun (Bard, Magnum Biopsy Instrument, Covington, Georgia, USA) or a semiautomated biopsy gun (PRECISA, Hospital Service, Aprilia, Italy). We obtained a minimum of three samples for each biopsy. Eleven patients, presenting multiple lesions in the context of the duct, directly underwent surgery because of the slight risk of association between papilloma and papillomatosis with breast cancer [24].

### 2.5. Statistical Analysis

Diagnostic sensitivity, specificity, and positive and negative predictive value (PPV and NPV) for both modalities were assessed.

The ROC analysis tested the galactography and MRI diagnostic ability separately. Subsequently, they were compared to histological analysis.

The statistical ROC analysis method is commonly used to evaluate the efficacy of a single diagnostic modality. The combined use of different complementary modalities makes it necessary to evaluate the single contribution provided by each of these modalities.

The area under the curve (AUC) of each diagnostic modality was calculated and results were presented with 95% of confidence intervals (CI 95%).

We performed Fisher's exact probability test for univariate analyses and, where possible, the *χ* ^2^ test with continuity correction.

Statistical analysis was performed using SPSS 19.0 for Windows and statistical significance was set at *P* < 0.05.

## 3. Results

Histologic examination performed after surgery or CNB showed that our sample was composed by 23 patients with papillomas (43%), 11 with papillomatosis, (21%) 5 with DCIS (10%), and 6 with papillary carcinomas (11%). Eight patients (15%) without lesions both on galactography and on MRI follow-up were classified as negative.

Considering the benign lesions, 2/23 cases with histological diagnosis of papilloma were classified as G2 and 13/23 as G3 through galactography according to Gregl classification. These cases were considered as false negative (FN), G2 being a normal finding and G3, the condition of ductal ectasia, a nonspecific finding. In the remaining eight cases galactography was diagnostic, while MRI correctly identified all the lesions (19/23 cases of mass-like enhancement, 2/23 cases of ductal enhancement, and 2/23 cases of the association of mass-like and ductal enhancement). In 10 cases, even with precontrast sequences we observed solid intraductal formation.

Concerning papillomatosis, galactography correctly identified the pathology in 5/11 cases, while it classified as G3 (nonspecific findings) 6/11 cases of papillomatosis, which were considered as FN. MRI was diagnostic in all cases, showing 8 ductal and 3 regional enhancements. Moreover, through precontrast sequences, we identified three cystic ductal ectasia cases and two solid intraductal mass cases.

Concerning malignant disease, 6 patients had papillary carcinoma; MRI correctly identified all of them (2 regional enhancement cases, 4 ductal enhancement cases, 2 simple and 2 branched enhancement, each one of them was associated with ductal ectasia), while galactography showed one FN case.

Considering DCIS, galactography and MRI correctly identified 4/5 cases; one of them was classified as G2 through galactography and showed no contrast medium uptake through MRI, so it was considered as FN.

Overall, we did not observe false positive (FP) cases; MRI showed one FN case (one case of DCIS) and galactography 23 FN cases (15 cases of papilloma, 1 case of papillary cancer, 1 case of DCIS, and 6 cases of papillomatosis). Galactography showed an overall sensitivity of 48.89%, a specificity of 100%, a PPV of 100%, and a NPV of 25.81% in detecting the presence of ductal pathologies, while for MRI sensitivity was of 97.78%, specificity of 100%, PPV of 88.89%, and NPV of 100%.

Once we evaluated capability of both methods to identify the ductal pathology, we tried to establish whether there was a specific radiological sign associated with a specific histologic subtype, in order to perform a correct differential diagnosis between benign and malignant lesions.

Considering galactography, the univariate analysis showed a statistically significant association (*P* < 0.001) between ductal distortion (G6) and papillary cancer and between filling stop (G5) and DCIS (*P* = 0.034) (Table 1). 35/53 cases presented ductal ectasia (G3), thus resulting in a nonspecific finding. The filling defect (G4) has been the most frequent finding in papilloma cases (7/23), but these data were not significant.

**Figure 2:**
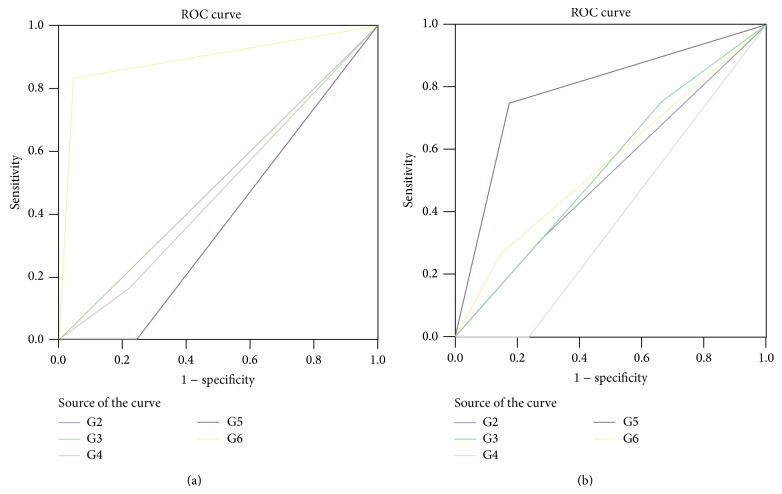
The ROC curves show an association between (a) papillary cancer and G6 on galactography (AUC 0.894; CI 0.715–1.074) and (b) between DCIS and G5 (AUC 0.790; CI 0.534−1.046).

The ROC analysis allowed us to verify our data, demonstrating an association between G6 at galactography and papillary cancer (AUC: 0.894; CI 0.715–1.074) and between G5 and DCIS (AUC: 0.790; CI 0.534−1.046), as shown in Figures 2(a) and 2(b).

In addition, galactography showed an overall trend to correctly identify malignant lesions, but no significant association has been found between one Gregl scale radiological sign and histological benign lesions. Considering MRI, we found a statistically significant association between mass enhancement and the presence of papilloma (*P* < 0.001), between ductal enhancement and papillomatosis (*P* < 0.001), between segmental enhancement and DCIS (*P* = 0.007), and between linear enhancement and papillary cancer (*P* = 0.011) as shown in Table 2.

**Figure 3:**
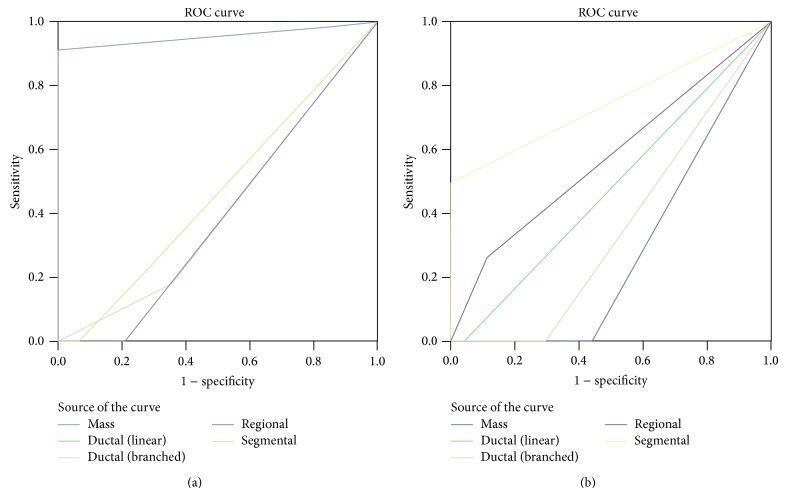
ROC analysis confirms the association between (a) mass enhancement and papilloma (AUC 0.957; CI 0.888−1.025) and (b) segmental enhancement and DCIS (AUC 0.750; CI 0.429−1.071).

ROC analysis confirmed the association between mass enhancement and papilloma (AUC 0.957; CI 0.888−1.025), segmental enhancement and DCIS (AUC 0.750; CI 0.429−1.071), and ductal enhancement and papillomatosis (AUC 0.790; CI 0.623–0.958), as shown in Figures 3(a) and 3(b).

## 4. Discussion

Nipple discharge is a relatively common symptom in the clinical practice and in most cases it is related to a benign condition. However, if it is especially spontaneous and shows a bloody/serous-bloody content, it requires a careful analysis to exclude malignant diseases. Therefore, it is important not only to identify the presence of disease but also to discriminate the causes of the discharge, differentiating malignant from benign lesions.

In this study we attempted to evaluate the role of the two imaging methods, galactography and MRI, in a patients' sample presenting a specific symptom, ductal bloody and serous-bloody discharge.

We found a statistically significant difference in overall sensitivity between the two methods. Particularly, MRI showed higher value of sensitivity (97.78% versus 48.89% galactography sensitivity) to identify ductal pathologies, while for both the methods specificity was 100%. In our analysis, the galactography sensitivity rates were lower than the others previously reported [25, 26]. This is probably due to the fact that in these previous studies the ductal ectasia (DE) condition had been considered as a pathologic finding. However, DE is also very common in asymptomatic patients, who never presented nipple discharge. In our sample, ductal ectasia has been found in 49/53 patients and particularly in all patients without disease. Therefore, we considered G3 as a pathological finding only if it is associated with other ductal anomaly signs.

Considering the capability to make a correct disease interpretation, even if the two methods specificity was the same (100%), MRI provided imaging findings, which allowed us to understand the underlying disease causing the pathologic discharge. In fact, we could see not only the pathological duct or ducts, using T2 and T1 precontrast sequences, but also the surrounding parenchyma enhancement. This aspect gave the overall evaluation of disease, justifying the MRI high sensitivity.

The breast MRI role in evaluating nipple discharge is still controversial [21, 22, 27]. Current indication for breast MRI, according to EUSOBI guidelines, does not include the evaluation of patients presenting with nipple discharge, although the potential role of MRI has already been evaluated in previous studies [28].

Several authors have carried out studies on the role of MRI contrast galactography cannulating and using intraductal contrast materials to enhance the secreting ducts. In 1997 Yoshimoto et al. [29] performed MRI contrast galactography after galactography in a patient, and the contrast material was injected both intravenously and into the discharging duct. Yücesoy et al. [30] compared conventional galacto-graphy with MRI contrast galactography in a prospective study performed with 16 patients showing 81% concordance between the two methods; their data suggest that MRI contrast galactography could be used as an alternative imaging modality for the pathologic nipple discharge diagnosis.

To the best of our knowledge, our study involved the largest sample in which all participants performed both galactography and conventional breast MRI using a breast dedicated coil without cannulating the secreting duct.

We evaluated if breast MRI could be used as an alternative imaging method in this specific group of patients, especially to express a differential diagnosis between benign and malignant lesions.

Galactography is a widely used and accepted diagnostic tool for visualizing and localizing ductal pathologies but frequently yields nonspecific findings, such as ductal ectasia, filling defects, and duct wall irregularities, which are ductal disease signs but does not always allow identification of the discharge cause. Thus, a positive study does not differentiate between malignant and benign discharge causes, and a negative study does not exclude an underlying carcinoma (or high-risk lesion). In our study the NPV for galactography was only 25.81% versus 100% for MRI [15, 29].

In addition, cannulation is possible only if the duct is discharging during the investigation time.

**Figure 4:**
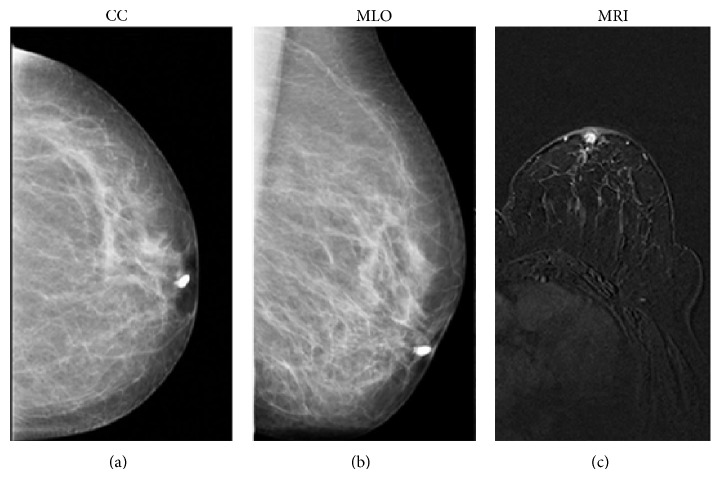
53-year-old patient presenting serous-bloody nipple discharge. Craniocaudal (a) and mediolateral oblique (b) mammographic images show the filling stop few millimeters distant from the nipple of the cannulated duct, imaging finding suggestive of papilloma. MRI image (T1-weighted 3D flash acquired in the axial plane) shows a mass intraductal enhancement in the retroareolar area (c) with round and sharp margins, indicative of a benign proliferation of the ductal epithelium (papilloma). Histology later confirmed the papilloma diagnosis.

If we consider benign diseases, in our sample galactography did not correctly identify 15 cases of papilloma and six of papillomatosis. On ductography, an intraductal papilloma appears as a round or lobulated filling defect, with a localized smooth surface, in a dilated duct (Figures 4(a) and 4(b)). The lesion typically arises from a single major duct in the subareolar region [31, 32]. To our experience, most lesions had small sizes (diameter less than 8 mm) non determining a complete duct obstruction. In addition, some lesions had a peripheral location; therefore, we could not observe direct radiological signs on galactography, which in most cases revealed only ductal ectasia. Instead, all benign lesions were correctly identified through MRI. Particularly, papillomas were detected when galactography was unsuccessful, appearing as an enhancing mass in a dilated duct, as shown in Figure 4(c).

**Figure 5:**
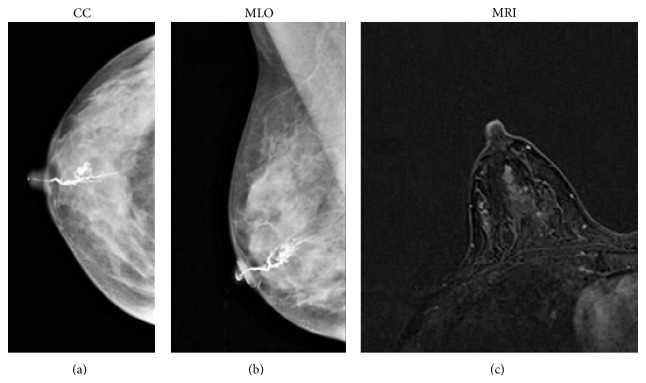
41-year-old patient with persistent bloody discharge of the right breast: craniocaudal (a) and mediolateral oblique (b) galactographic projections show a ductal ectasia condition with wall duct focal irregularities. MRI images (T1-weighted 3D flash), acquired on the axial plane, show a ductal enhancement. Histology revealed a papillomatosis condition.

**Figure 6:**
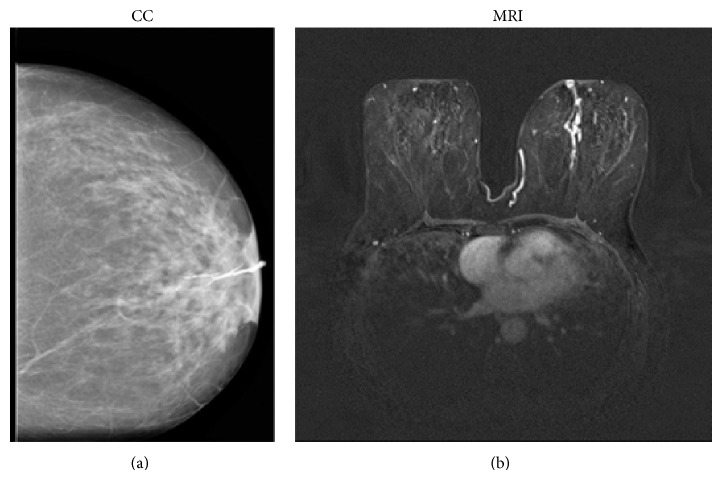
54-year-old patient, presenting with bloody nipple discharge from the left breast: craniocaudal galactographic image (a) shows ductal ectasia and filling defect, as observed in papillomatosis. (b) MRI images (T1-weighted 3D flash), acquired on the axial plane, show a branched inhomogeneous ductal enhancement, suspicious of ductal malignant pathology. Histology revealed DCIS. This case represents a galactography FN case; MRI correctly identified the malignant* disease*.

We found a statistically significant association between papillomatosis (Figures 5(a), 5(b), and 5(c)) and ductal enhancement. Ductal irregularity, encasement, distortion, obstruction, or irregular filling defects are conventional galactography findings suggestive of malignancy. We found a statistically significant association between ductal distortion (G6) and papillary cancer and between “stop” (G5) and DCIS. Galactography was able to discriminate malignant lesions; in two FN cases, it revealed only a modest ductal ectasia without filling defects or wall irregularities (Figures 6(a) and 6(b)).

In our study, MRI correctly identified malignant lesions. It showed only one false negative case: a patient with DCIS who was not identified with galactography. Although this patient has been classified as negative both on galactography and on MRI, surgery was performed because of a persistent discharge after one month follow-up and of a positive cytology showing epithelial cells. Wenkel et al. [22] found the same results; they had a DCIS case in a patient with bloody nipple discharge not identified on MRI. Our patient had a very small (7 mm), low-grade DCIS, which was not showed at MRI.

Nonetheless, our study has several limitations. The first one is due to its retrospective design. Patients were selected because of a specific symptom with a high possibility to have pathology (benign or malignant); in fact, the majority of them (45/53) had disease. This caused for both methods a high specificity value. In addition, we had a restricted sample, because we excluded a large number of patients with nipple discharge who did not perform galactography for nipple retraction or intermittent discharge. Furthermore, the follow-up period was limited: in patients who did not undergo biopsy or surgery, the mean follow-up was only of 18 months. An optimal follow-up should be at least three years to assess the actual imaging examination false-negative rate.

## 5. Conclusions

Cancer represents a significant risk to patients having suspicious bloody/serous-bloody discharge. Even if ductography represents the standard imaging technique for these patients evaluation, it is not always available and the possibility to differentiate between benign and malignant lesions may be controversial. Our findings suggest that MRI is able to differentiate between the nipple discharge causes and according to the reported literature, it shows a high sensitivity value in detecting the ductal disease. Therefore, nipple discharge should be considered a valid indication to perform MRI.

## Figures and Tables

**Table 1:** Univariate analysis showing galactography compared to histological finding.

Histology	G2	G3	G4	G5	G6
	*n* (%), *P*	*n* (%), *P*	*n* (%), *P*	*n* (%), *P*	*n* (%), *P*
No pathology	**7 (63.7), 0.001**	**2 (5.9), 0.019**	0, 0.175	0, 0.174	0, 0.578
Papilloma	2 (18.2), 0.089	18 (52.9), 0.113^*^	7 (58.3), 0.392^*^	6 (50), 0.899^*^	**0, 0.016**
Papillary cancer	1 (9.1), 1	4 (11.8), 1	1 (8.3), 1	0, 0.316	**5 (71.4), <0.001**
DCIS	1 (9.1), 1	3 (8.8), 1	0, 0.577	**3 (25), 0.034**	1 (14.3), 0.530
Papillomatosis	0, 0.94	7 (20.6), 1	4 (33.3), 0.244	3 (25), 0.701	1 (14.3), 1

^*^ *χ* ^2^ test with continuity correction.

**Table 2:** Univariate analysis showing MRI compared to histological finding.

Histology	Mass	Ductal (linear)	Ductal (branched)	Regional	Segmental
	*n* (%), *P*	*n* (%), *P*	*n* (%), *P*	*n* (%), *P*	*n* (%), *P*
Negative	0, 0.15	0, 1	0, 0.093	0, 0.573	0, 1
Papilloma	**21 (95.5), <0.001** ^*^	0, 0.499	4 (28.6), 0.225	**0, 0.028**	0, 0.499
Papillary cancer	**0, 0.035**	**2 (100), 0.011**	2 (14.3), 0.649	2 (33.3), 0.136	0, 1
DCIS	1 (4.5), 0.389	0, 1	0, 0.309	1 (16.7), 0.397	**2 (100), 0.007**
Papillomatosis	**0, 0.001**	0, 1	**8 (57.1), <0.001**	3 (50), 0.101	0, 1

^*^ *χ* ^2^ test with continuity correction.
